# Dehulled small millets: The promising nutricereals for improving the nutrition of children

**DOI:** 10.1111/mcn.12791

**Published:** 2019-05-31

**Authors:** Malathi Durairaj, Gurumeenakshi Gurumurthy, Varadharaju Nachimuthu, Karthikeyan Muniappan, Subbulakshmi Balasubramanian

**Affiliations:** ^1^ Department of Post Harvest Technology Centre Tamil Nadu Agricultural University Coimbatore India; ^2^ Small Millet Foundation DHAN Foundation Madurai India

**Keywords:** anthropometric, child nutrition, processing equipment, small millets, supplementation

## Abstract

Good nutrition during a child's early years lays a strong foundation of health for the rest of its life. Yet in India, there is widespread prevalence of undernourishment among children below 5 years of age. Within the Indian context, small millets have great potential as a healthy food to address this challenge by the virtue of their nutritional qualities. However, there are many problems with the current processing technology for small millets, whereas the use of value‐added products was minimal. To address this, an assessment of existing small millet processing machinery was undertaken, and a double chamber centrifugal dehuller was developed, which had higher recovery of dehulled unpolished millets and met requirements at the village and enterprise levels. To demonstrate the health benefits of consuming value‐added small millets, a study of supplementation of multi‐millet health mix on the nutritional status of primary schoolchildren was conducted in Thondamuthur Block of Coimbatore District, India. Multi‐millet health mix was formulated from kodo millet, little millet, foxtail millet, finger millet, and wheat with the inclusion of pulses. It contained 65.45‐g carbohydrate, 11.46‐g protein, 4.94‐g fat, 4.94‐g fibre, 4.07‐mg iron, 112‐mg calcium, 268.52‐mg phosphorus, and 349 calories of energy per 100 g. The study indicated that there was a significant increase in height, weight, and haemoglobin level of the schoolchildren who regularly consumed the formulated multi‐millet health mix. The improved huller and value‐added food product developed can be feasible options for improving nutrition security and livelihoods through increased use of small millets.

**Key messages**

- The improved huller prototype developed for small millets increased the millet rice recovery by more than 10% and met processing requirements at village and enterprise levels.
- Multi‐millet health mix formulated retained nutrient content in appreciable amount and has a positive effect on the nutrition of primary schoolchildren.
- Disseminating the improved huller and multi‐millet health mix can help in improving nutrition security and livelihoods.

## 1 INTRODUCTION

Millets are important food crops in developing countries and recognized as high‐energy nutritious food that can help in reducing malnutrition, nourishing the population, and preventing and curing lifestyle‐related health issues such as diabetes, cardiovascular diseases, and obesity. Small millets, a subset of millets, offer better nutrition with various micronutrients, high protein, high dietary fibre, and low glycemic index when compared with mainstream cereals like paddy rice and wheat. They have a well‐balanced amino acid profile and are good sources of calcium, phosphorous, and iron. India leads in the production of many of the small millets. Within the Indian context, these include finger millet, kodo millet, little millet, foxtail millet, proso millet, barnyard millet, and browntop millet (Saleh, Zhang, Chen, & Shen, 2013; Sarita & Singh, 2016).

Malnutrition during childhood has serious future health‐related implications. Many studies have shown a strong relationship between fetal malnutrition and early‐life exposure to obesity and type 2 diabetes (Praveen & Tandon, 2016). In India, children have been the most affected demographic of the undernourished population. Nearly every third child is considered undernourished, with the prevalence rate of underweight, stunted, and wasted among the children under 5 years hovering at 35.7%, 38.4%, and 21%, respectively, whereas every second child is anaemic (58.4%; Government of India, 2017).

Owing to the cultivation and nutritional significance of small millets in India, research outlined in the subsequent sections was undertaken with two primary purposes. One focused on the development of improved millet processing equipment, and the other focused on value addition through developing multi‐millet health mix and studying the effect of its supplementation on the growth of primary schoolchildren.

## 2 METHODS

### 2.1 Assessment of existing small millet processing machineries

Structured assessment of existing small millet processing machineries was carried out with a team of experts, equipment manufacturers, and processors (users). The machineries assessed included the grader, huller, and destoner. The assessment was made in terms of efficiency and “ease of use.”

### 2.2 Development of double chamber centrifugal dehuller

Dehulling is one of the important processing steps carried out for the removal of husk from millets. A double chamber centrifugal dehuller was developed, whereby it essentially consists of a feed hopper, two centrifugal chambers made of cast iron, impellers with curved vanes, blower, and separate outlets for collecting kernel and husk. The unit is powered by 5 hp motor with a suitable power transmission system. An elevator is also provided for easy feeding of the grains into the unit. In operation, the grains from the hopper enter the impeller through feed housing, where it gains momentum and thrown against the cast iron chamber at a very high velocity. The splitting of the husk occurs due to impact, and the kernel is separated from the husk. The husk and kernel mixture passes through a chamber, where they are segregated by means of a blower provided at the bottom of the unit.

### 2.3 Development of multi‐millet health mix and its effect on nutritional status of primary schoolchildren

As part of the efforts to realize the potential of small millets to address nutrition deficiencies, the development of value‐added product and the study of its health benefits were undertaken, in addition to addressing the challenges related to primary processing. Feeding trials were carried out to understand the effect of value‐added small millets on the nutrition of primary schoolchildren.

#### 2.3.1 Development of multi‐millet health mix

##### Formulation of multi‐millet health mix for supplementation

The dehulled millets obtained from centrifugal dehuller were used for the development of a multi‐millet health mix, where the dehulled millets were pulverized into flour. On the basis of the previous work conducted, the nutrient dense multi‐millet health mix was formulated using the ingredients of millet flour (finger, kodo, little, and foxtail millets), wheat, pulses (greengram, bengalgram, soya bean, and peas), groundnut, nuts (almonds and cashew), milk powder, jaggery, and flavouring agents (cardamom and dried ginger). These millets were chosen given the ease of digestibility of their starch. Furthermore, the selected millets also have high protein (10–12%) and minerals (5–8%), particularly calcium, phosphorus, magnesium, and iron, which are essential for the growth and development of children. From the previous trials conducted where in the quantity of millet added varied from 50 to 300 g, it was concluded that the addition of 200 g of each of the small millets was ideal for the multi‐millet health mix in terms of consumer acceptability. Pulses selected for this study contained protein of high biological value. Groundnut was added for its calorific value and jaggery for its iron content. The combination of all these ingredients contributes to quality and quantity of nutrients in the mix and makes the supplement a suitable food for improving the health status of primary schoolchildren. The amount of ingredients incorporated in the multi‐millet health mix is presented in the Table 1.

**Table 1:** Ingredients incorporated in the multi‐millet health mix

S. no.	Items	Quantity and share, g (%)
1	Wheat	200 (13)
2	Kodo millet	200 (13)
3	Little millet	200 (13)
4	Foxtail millet	200 (13)
5	Finger millet	200 (13)
6	Greengram	200 (13)
7	Whole bengalgram	20 (1.3)
8	Whole soya beans	20 (1.3)
9	Jaggery	200 (13)
10	Peas (dried)	20 (1.3)
11	Groundnut	20 (1.3)
12	Almonds	20 (1.3)
13	Cashew	20 (1.3)
14	Dried ginger	5 (0.3)
15	Cardamom	2 (0.1)
16	Milk powder	20 (1.3)

All other ingredients except jaggery and milk powder were carefully roasted to enhance the flavour and texture and then finely powdered. Roasting was used because it partially breaks down the starch and facilitates easy digestion. Additionally, it helps to minimize the anti‐nutritional factors in the pulses. Roasting of the flour before pulverizing increases the shelf life of the multi‐millet health mix and gives a thin textured gruel or porridge, which has led to increased acceptability by the children. Therefore, roasting is considered an essential process in the preparation of multi‐millet health mix targeted for children. Subsequently, jaggery and cardamom powder were mixed, and the final prepared multi‐millet health mix was packed in high‐density metallized polypropylene packs and stored in ambient condition.

For the preparation of the porridge, 25 g of the multi‐millet health mix was taken, and 60 ml of water was added to it. It was then heated for 15 min for complete gelatinization of starch to complete the cooking. The net content obtained after 15 min of cooking is 75 ml of porridge. As per Indian Council of Medical Research, the recommended amount of porridge to be given to a child at a time is 75 ml.

##### Nutrient content of multi‐millet health mix

The moisture, carbohydrates, protein, fat, fibre, ash, calcium, and iron were determined by the AOAC (2000).

#### 2.3.2 Effect of supplementation of multi‐millet health mix on nutritional status of primary schoolchildren in the age group of 4–6 years

##### Study area and subjects

Total attendance of children in the age group of 4–6 years in the primary school at Thondamuthur Block of Coimbatore District, India, was 200. Out of them, 60 children were selected as subsamples for further in‐depth study through purposive sampling, which involved getting parental authorization after explaining the purpose of the study (ethical clearance obtained from competent authorities).

##### Assessing the effect of multi‐millet health mix on nutritional status of the selected study groups

The selected 60 primary schoolchildren in the age group of 4–6 years were divided equally into control and experimental groups. There was no nutritional supplementation provided for the control group. The experimental group of primary schoolchildren received 25 g of multi‐millet health mix in the form of porridge (75 ml) daily by mid‐morning for a period of 6 months. The nutritional status of both control and experimental groups was determined with the help of anthropometric measurements of height and weight, and biochemical indices of haemoglobin level, before and after supplementation. Following the standard paired *t* test given by Rangaswamy (1995), the mean height, weight, and haemoglobin level of the experimental group were compared with that of the control group.

## 3 RESULTS AND DISCUSSION

### 3.1 Assessment of existing processing machineries

A structured assessment of the existing processing machineries by a team composed of equipment developers, equipment manufacturers, and users was carried out with the subsequent identification of areas of improvement to enhance the quantity and quality of output and to improve “ease of use” (Karthikeyan et al., 2016). In general, small millet dehulling was carried out with the abrasive roller type machines, as exclusive dehuller for the small millets was not available. In this process, along with the husk, bran and small portion of endosperm were also removed, which leads to the depletion of nutrients. Moreover, the dehulled grains contain about 20% of brokens. Hence, a dire need for the development of a dehuller for small millets that removes only the husk and not the bran. The dehuller should also have higher recovery of kernels with minimum breakage. The hulling technology needed to be optimized for hulling various small millets, which differ from one another in terms of size, hardness, and shape. The separation mechanism in the huller needed to be improved to reduce the removal of grits and other usable materials along with husk. The post‐hulling machinery had to be improved for better separation of unhulled and hulled grains and to separate finer stones and mud balls similar in size and weight from millet rice and grits. The scale of the huller also had to be increased to meet the processing requirements at the small and medium enterprise (SME) level. Finally, the grader needed to be improved to meet pre‐ and post‐hulling segregation requirements of different small millets and to reduce the machine's footprint.

### 3.2 Double chamber centrifugal dehuller

The comparative recovery of dehulled unpolished millets by abrasive type of dehuller and double chamber centrifugal dehuller is given in Table 2. From Table 2, it can be inferred that the efficiency of kernel recovery is higher in centrifugal dehuller by 10% than in the abrasive huller irrespective of the small millets. The other special features of the developed dehuller are as follows: (a) it is suitable for all small millets that need hulling and (b) the breakage is less than abrasive type of dehuller (4–5%). The dehuller was also versatile in terms of scale of operation, which varied from 50 to 500 kg/hr and met the requirements at the village, micro enterprise, and SME levels. The dehulled small millets retained major portions of bran and endosperm, thereby retaining the vitamins, minerals, and the soluble fibre in the dehulled millets. Hence, dehulling in the developed dehuller yielded a product, which is nutritionally superior to conventionally milled small millets and is considered suitable for the development of health foods.

**Table 2:** Recovery of kernel from millets (100 kg of grain)

Small millets	Available kernel (with bran), kg	Abrasive type of dehuller, kg	Double chamber centrifugal dehuller, kg	Efficiency %	Breakage %
Little millet	78 (6)	65	75	95.5	4.5
Proso millet	79 (6)	66	75.5	96	4
Foxtail millet	75 (6)	64	72	95	4.5
Barnyard millet	68 (7.5)	56	65	95.5	4.5
Kodo millet	67 (8)	55	64	95	5

### 3.3 Development of multi‐millet health mix

The organoleptic qualities, namely, colour, appearance, taste, and flavour, and overall acceptability of multi‐millet health mix were assessed by 30 panellists by rating on a 9‐point hedonic scale (9 as *like extremely* and 1 as *dislike extremely*) as outlined by Watts, Ylimaki, Jeffery, and Elias (1989). The results revealed that the product was highly acceptable to the panel members. The results also indicated that it would be highly acceptable to the selected subjects. The multi‐millet health mix contained 16.36‐g carbohydrate, 2.87‐g protein, 1.24‐g fat, 1.24‐g fibre, 1.02‐mg iron, 28‐mg calcium, 67.13‐mg phosphorus, and 87 calories of energy per 25 g.

The developed multi‐millet health mix had a shelf life of 180 days at ambient conditions. The nutritional quality of the multi‐millet health mix was maintained throughout the storage period with a slight reduction in starch, protein, and fat. Shanthi, Manimegalai, and Chitra (2000) reported that instant idli mix had an initial protein content of 11.37 g per 100 g, which was gradually lost upon storage, and the loss was reported to be 1.4 g per 100 g. However, in the present study, the loss of protein was found to be comparatively less (0.17 g per 100 g). This might be due to the packaging material, which was metallized polypropylene and the storage condition. Premavalli, Majumdar, Madhura, and Bawa (2003) also reported a decrease in moisture content and other proximate compositions with increase in incorporation of finger millet flour in convenience mixes. A slight increase in moisture was reported on storage of composite mix based on millets during a storage period of 6 months in polyethylene bags by Itagi, Naik, and Shanthkumar (2003), but the same mix did not show any moisture pick up when packed in metallized polypropylene covers. These findings concur with those of the present research findings. Meenatchisundaram (2005) observed that the fibre and tannin content of the pearl millet‐based convenience mix showed a non‐significant change during storage. Similar observations were also recorded in the present study.

### 3.4 Assessing the effect of multi‐millet health mix on nutritional status of the selected study group

#### 3.4.1 Mean height and weight of the selected subjects

The mean height and weight of the selected primary schoolchildren are presented in the Table 3. It can be observed that there was significant improvement in mean height of children due to 6 months of supplementation with multi‐millet health mix. The mean height of the children increased by 2.82 cm in the experimental group, whereas in the control group, it only increased by 1.03 cm. Significant improvement was also observed in the case of mean weight, which increased by 2.64 kg in the experimental group compared with 1.43 kg in the control group.

**Table 3:** Effect of supplementation with multi‐millet health mix on mean height and weight of the selected primary schoolchildren

Parameters	Experimental group				Control group			
	Mean ± *SD*		Mean difference	*t* value	Mean ± *SD*		Mean difference	*t* value
	Initial	Final			Initial	Final		
Height (cm)	106.124 ± 7.51	108.944 ± 5.62	2.82	10.625*	106.305 ± 0.17	107.335 ± 7.53	1.03	3.21^NS^
Weight (kg)	15.88 ± 2.82	18.52 ± 3.14	2.64	11.251*	15.30 ± 1.61	16.73 ± 1.92	1.43	2.03^NS^

*Note*. NS: not significant.

* Significant at 5% level.

#### 3.4.2 Haemoglobin level of the selected subjects

Haemoglobin level of the selected subjects in the study groups before and after supplementation was recorded and presented in the Table 4. It was noted that there was an increment of haemoglobin level in the experimental group as compared with control, indicating that supplementation of millet–pulse‐based health mix improved haemoglobin level in primary schoolchildren.

**Table 4:** Effect of supplementation with multi‐millet health mix on mean value of the sample haemoglobin level of the selected primary schoolchildren

Parameter	Experimental group				Control group			
	Mean ± *SD*		Mean difference	*t* value	Mean ± *SD*		Mean difference	*t* value
	Initial	Final			Initial	Final		
Haemoglobin	8.71 ± 0.72	9.25 ± 0.82	0.54	6.85*	8.63 ± 0.21	8.89 ± 0.22	0.26	1.26^NS^

*Note*. NS: not significant.

* Significant at 5% level.

The supplementation studies proved that multi‐millet health mix has a positive effect in increasing the anthropometric indices of children. This might be because it was calorie rich and had the required carbohydrate, protein, and fat. This study is in line with the study done by Khader and Maheswari (2012), which found that there was significant increase in weight of preschool children after supplementation of amylase‐rich malted mixes for the period of 4 months.

## 4 CONCLUSION

The centrifugal dehuller developed had improved efficiency of dehulling, with minimum breakage. Dehulling in the developed dehuller yielded a product, which is nutritionally superior to conventionally milled small millet rice, as the major portion of bran and endosperm were retained. The developed dehuller met the processing requirements at the village, micro enterprise, and SME levels. It can be disseminated for adoption at the village and enterprise levels by the government as part of its development schemes for improving the availability of nutritious small millet products widely. Furthermore, supplementation of multi‐small millet‐based health mix resulted in the improvement in the nutritional status of the primary schoolchildren in terms of height, weight, and haemoglobin level. The developed multi‐millet health mix can be introduced in state nutrition programmes, as it will help in reaching the health benefits of small millets to the needy population on a scale. Thus, the improved small millet huller and value‐added food product developed have strong potential to improve the nutrition security and rural/tribal livelihoods.

## CONFLICTS OF INTEREST

The authors declare that they have no conflicts of interest.

## CONTRIBUTIONS

The partners of the study were Tamil Nadu Agricultural University, Coimbatore and DHAN Foundation, Madurai, India. The authors are the contributors for conducting the study and preparation of the manuscript. All authors have reviewed and approved the manuscript.
